# Bias in microRNA functional enrichment analysis

**DOI:** 10.1093/bioinformatics/btv023

**Published:** 2015-01-20

**Authors:** Thomas Bleazard, Janine A Lamb, Sam Griffiths-Jones

**Affiliations:** ^1^Faculty of Medical and Human Sciences, and ^2^Faculty of Life Sciences, University of Manchester, UK

## Abstract

**Motivation:** Many studies have investigated the differential expression of microRNAs (miRNAs) in disease states and between different treatments, tissues and developmental stages. Given a list of perturbed miRNAs, it is common to predict the shared pathways on which they act. The standard test for functional enrichment typically yields dozens of significantly enriched functional categories, many of which appear frequently in the analysis of apparently unrelated diseases and conditions.

**Results:** We show that the most commonly used functional enrichment test is inappropriate for the analysis of sets of genes targeted by miRNAs. The hypergeometric distribution used by the standard method consistently results in significant *P*-values for functional enrichment for targets of randomly selected miRNAs, reflecting an underlying bias in the predicted gene targets of miRNAs as a whole. We developed an algorithm to measure enrichment using an empirical sampling approach, and applied this in a reanalysis of the gene ontology classes of targets of miRNA lists from 44 published studies. The vast majority of the miRNA target sets were not significantly enriched in any functional category after correction for bias. We therefore argue against continued use of the standard functional enrichment method for miRNA targets.

**Availability and implementation:** A Python script implementing the empirical algorithm is freely available at http://sgjlab.org/empirical-go/.

**Contact:** sam.griffiths-jones@manchester.ac.uk or janine.lamb@manchester.ac.uk

**Supplementary information:** Supplementary data are available at *Bioinformatics* online.

## 1 Introduction

MicroRNAs (miRNAs) down-regulate abundance and translation of target mRNAs through complementary binding to target sites. miRNAs play important roles in regulating gene expression in response to stimuli and during development and their expression patterns can be predictive of disease states (Leidinger *et al*., 2013; Schratt *et al*., 2006; Xie *et al*., 2013). For this reason, a large number of studies have investigated the expression of miRNAs in a wide range of biological conditions. Microarray assays, qRT-PCR and high-throughput sequencing have all been used to identify differentially expressed miRNAs in disease states, between different tissues and during development (Davidson *et al*., 2010; Liang *et al*., 2013; Wu *et al*., 2012a). Unfortunately, the interpretation of miRNA differential expression is not straightforward. The roles of individual miRNAs in cellular pathways are still poorly understood. Each miRNA has the potential to target hundreds of different genes, meaning that perturbation of a single miRNA may affect many biological functions (Friedman and Farh, 2009). This motivates a broad view: given a list of differentially expressed miRNAs, we must look for the functions or pathways on which they converge.

Here, we examine the most common method of miRNA functional enrichment analysis, used in hundreds of published studies. This method consists of three steps: finding which genes are targeted by selected miRNAs, annotating target genes for their participation in pathways and processes, and statistical testing for over-representation of a biological process in the set of targeted genes (Gusev *et al*., 2007). For the first step, computational target prediction is usually necessary because experimental datasets covering miRNA–mRNA interactions on a genome scale are currently lacking. For the second step, annotation by gene ontology (GO) term membership or Kyoto Encyclopedia of Genes and Genomes (KEGG) pathways is common (Ashburner *et al*., 2000; Kanehisa and Goto, 2000). For the final step, the hypergeometric distribution, or equivalently Fisher's exact test, is used to test for enrichment. The hypergeometric distribution describes the situation where samples are picked uniformly at random from a finite population which contains a labelled subset. In the context of functional enrichment, it gives the probability of targeting *k* genes from a labelled category when targeting a total of *n* genes from the genome. We can then use this distribution to test the null hypothesis that genes were targeted randomly versus the alternative that genes belonging to a given annotation were preferentially targeted.

This approach, which we here refer to as the ‘standard method’ because of its preponderance, consistently produces a large number of significantly enriched processes. However, these are often difficult to interpret and full lists of significant terms are rarely provided in published articles. Recurrence of GO terms between apparently unrelated diseases and conditions in the literature is very notable (Lu *et al*., 2012). Most worryingly, even random and meaningless miRNA lists produce significant functional enrichments using the standard method (Ritchie *et al*., 2009).

In this study, we show that critical problems with the standard method arise because of bias in the sets of genes that are predicted to be targeted by miRNAs in general. This means that the assumption of uniform sampling in the hypergeometric distribution is not reasonable. We correct for this bias by bringing the statistical test back from the level of genes to the level of miRNAs and show that most functions reported as significantly enriched in the literature do not remain so after correction.

## 2 Methods

We developed an algorithm to find the empirical distribution of the number of miRNA target genes within annotated functional categories. We predicted targets of all miRBase release 20 annotated mature miRNAs in the 3′ UTRs of all Ensembl release 75 human and mouse genes using miRanda (version 3.3a, free energy < −20 kcal/mol, score > 155) (Enright *et al*., 2003). Annotated biological process GO terms for all human and mouse genes were downloaded from Ensembl (Ashburner *et al*., 2000; Kinsella *et al*., 2011). Following the standard method, we defined the target genes for a list of miRNAs as the union of genes predicted to be targeted by each miRNA. We then calculated GO term overlap as the proportion of target genes which were annotated as belonging to a given GO term. Our empirical algorithm first counted the GO term overlap for targets of an input miRNA list. A set of miRNAs of the same size as the input list was randomly sampled without replacement from the set of all annotated miRNAs, with one million iterations. An empirical *P*-value for each GO term was calculated using the proportion of simulations that produced an equal or greater GO term overlap. We also developed a modified multi-hit version of this algorithm that did not use the simple union of target genes, but instead gave each gene a score given by the sum of the number of predicted target binding sites for all input miRNAs. GO term overlap was then defined as the score for genes annotated with a given GO term divided by total score for input miRNAs. We repeated all analyses using KEGG pathways [accessed through the KEGG.db Bioconductor package, which archives KEGG data from March 15, 2011 (Kanehisa and Goto, 2000)] in place of GO terms. We also recalculated target predictions based on the intersection of genes returned by three alternative target prediction algorithms: PITA predictions from the PITA Targets Catalog version 6 (August 2008) based on mm9 and hg18 for mouse and human, respectively, with zero flank and all sites included (Kertesz *et al*., 2007); DIANA-microT-v4 predictions, which use miRBase annotated miRNAs and Ensembl 3′ UTR sequences (Reczko *et al*., 2012); and canonical seed matches between miRBase miRNAs and Ensembl 3′ UTR sequences as in (Bartel, 2009). We used miRBase alias data to match the names of miRNAs in downloaded prediction sets to their current annotations (Kozomara and Griffiths-Jones, 2011) and Ensembl gene synonyms to match gene names in the target sets to Ensembl GO classifications.

We investigated the effects of filtering miRNA target predictions for experimentally supported target sites, similar to the approach of miRFunction (Li *et al*., 2014). Thirty-six human and five mouse AGO pulldown CLIP-seq datasets were downloaded from starBase (Li *et al*., 2014). Target predictions from miRanda were mapped to genomic loci and filtered to include only those supported by at least one experimental dataset.

We also investigated the use of the standard method in studies of plant miRNAs. We predicted TIGR genome cDNA (OSA1R5) targets of miRBase-annotated rice miRNAs using psRNATarget with default parameters (Dai and Zhao, 2011). We matched the TIGR loci to GO annotations in tables downloaded from the agriGO database (Du *et al*., 2010).

We performed a literature survey to identify studies that followed the standard method. We used the search functions provided by Nature Publishing Group and Public Library of Science, as well as Google Scholar, with the search terms ‘gene ontology’ and ‘microRNA’ for mouse and human and ‘oryza’ and ‘microRNA’ for rice. Each article was manually checked to confirm that the standard method was followed. Lists of miRNAs reported to be differentially expressed (or otherwise flagged) were manually compiled from the retrieved articles. Where multiple lists of miRNAs were assessed in the same manuscript, we arbitrarily chose one list for testing. We define results as significant by default where α < 0.05. In the case of multiple testing, we perform Benjamini–Hochberg adjustment and report significant items passing the threshold for false discovery rate < 0.05 (Benjamini and Hochberg, 1995).

## 3 Results

### 3.1 Assessing the appropriateness of the hypergeometric distribution

**Fig. 1.**
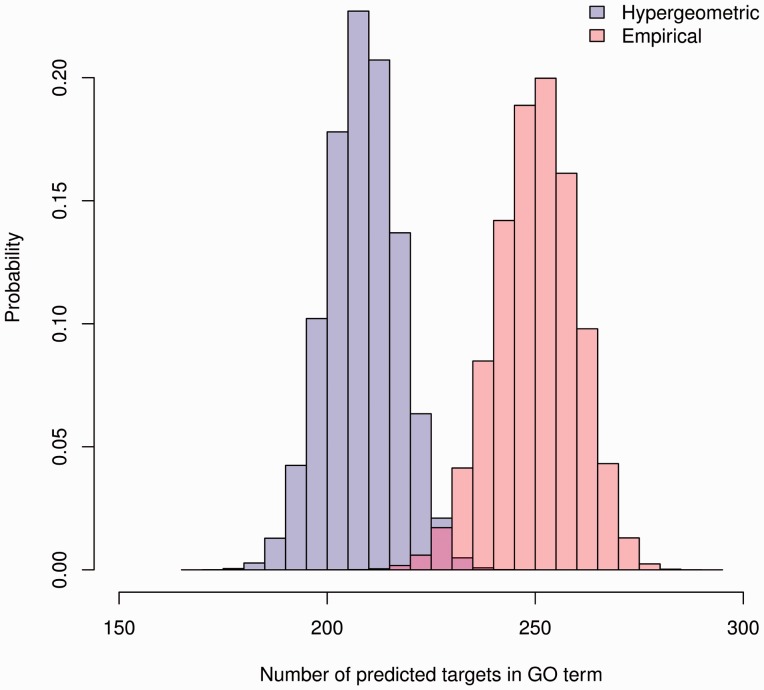
Expected and empirical number of predicted targets of randomly selected microRNAs. For an example 39 miRNAs, we calculate the hypergeometric distribution (blue) for the number of expected targets in the GO term ‘ion transport’ (GO:0006811). The empirical distribution (red) represents the predicted targets of random samples of 39 miRNAs. The probability for each 5-gene bin is given according to both distributions

We used our algorithm to investigate whether the null hypothesis used by the standard method was appropriate by comparing the hypergeometric distribution with an empirical distribution for the number of predicted target genes belonging to a GO term for randomly sampled miRNAs. As an illustration, we use the GO term ‘ion transport’ (GO:0006811), which is often reported as significantly enriched in the literature (Liu *et al*., 2010; Sokolov *et al*., 2012; Yunta *et al*., 2012). We predicted the targets of an example set of 39 miRNAs that were reported as differentially expressed in one study (Sokolov *et al*., 2012). These miRNAs were predicted to target 10 057 genes out of 15 733 genes with at least one assigned biological process GO term, of which 327 are annotated for ion transport. These parameters were chosen to mirror the methods used by the popular tool DAVID (Huang *et al*., 2009). The expected distribution of the number of target genes assigned to the ‘ion transport’ GO term according to the hypergeometric distribution is shown in Figure 1, alongside the distribution of the number of target genes of 1 million randomly chosen sets of 39 miRNAs. The data clearly show that the hypergeometric distribution does not adequately model the empirical background distribution under these conditions.

Figure 1 immediately suggests an explanation for the excess of significant GO terms under the standard method. A typical miRNA target gene set, with GO membership near the mean of the empirical distribution, will produce significant *P*-values for GO term enrichment using the standard method. Indeed, the mean number of targets involved in ion transport for 39 random miRNAs (250 genes) gave a *P*-value of 5.97 × 10^−^^7^ when tested using the hypergeometric distribution. We compared the hypergeometric with the empirical distributions for all biological process GO terms. For each GO term, we constructed the hypergeometric distribution using the same example of 39 miRNAs targeting 10 057 genes as predicted by miRanda, again mirroring a common DAVID analysis (Enright *et al*., 2003; Huang *et al*., 2009). For each GO term, we then generated an empirical distribution for the number of member genes targeted by 39 randomly selected miRNAs. Supplementary Table S1 lists the one-sided *P*-value on the hypergeometric distribution of the mean of the empirical distribution for each GO term. Smaller *P*-values represent terms that are more likely to be erroneously reported as significantly enriched using the standard method. Among the terms yielding the smallest *P*-values (Table 1), several are notably often reported as enriched in the literature, such as ‘regulation of transcription, DNA-dependent’ (GO:0006355) (Kraemer *et al*., 2013; Mizuguchi *et al*., 2011; Munch *et al*., 2013; Ziats and Rennert, 2013).

**Table 1.** GO terms with the largest difference between hypergeometric and empirical background distributions

GO term	Hypergeometric *P*-value of empirical mean
GO:0006351∼transcription, DNA-templated	1.21 × 10^−28^
GO:0006355∼regulation of transcription, DNA-dependent	6.99 × 10^−25^
GO:0007165∼signal transduction	4.63 × 10^−18^
GO:0006468∼protein phosphorylation	4.34 × 10^−17^
GO:0055085∼transmembrane transport	1.39 × 10^−13^
GO:0015031∼protein transport	1.95 × 10^−13^
GO:0045944∼positive regulation of transcription from RNA polymerase II promoter	3.44 × 10^−12^
GO:0045893∼positive regulation of transcription, DNA-dependent	4.80 × 10^−12^
GO:0048011∼neurotrophin TRK receptor signaling pathway	7.67 × 10^−12^
GO:0007264∼small GTPase mediated signal transduction	1.22 × 10^−11^

Using 39 miRNAs targeting 10 057 genes, we calculated the *P*-value on the hypergeometric distribution for the rounded mean of the empirical distribution for each GO term. The 10 processes with the most extreme bias are shown.

Other GO terms suffered from an opposite bias, making detection of a significant enrichment almost impossible under the standard method. The most extreme of these with more than five members were ‘defense response to bacterium’, ‘detection of chemical stimulus involved in sensory perception of smell’ and ‘G-protein coupled receptor signaling pathway’, with *P*-values close to 1. These terms were conspicuously absent from published lists of enriched processes in disease. These results imply that using the hypergeometric distribution to model miRNA target gene GO term membership is inappropriate and is liable to produce spurious results.

### 3.2 Re-analysis of published miRNA lists

We investigated the prevalence of the standard method with a non-exhaustive manual search of journal articles. We identified 40 published studies where the standard method was applied to investigate functional enrichment of targets of sets of animal miRNAs (Supplementary Table S2). Among these, a wide range of algorithms and their combinations were used for target prediction. Six different organisms were represented in studies ranging from sea cucumber aestivation to pseudorabies virus in pig cell lines (Chen *et al*., 2013a; Wu *et al*., 2012b). A large number of different web servers provided tools for functional annotation and statistical testing of enrichment. The types of functional categories tested included biological process GO terms with various filters and KEGG pathways (Huang *et al*., 2009). The list included recent and high-impact publications (e.g. Soh *et al*., 2013). Although functional enrichment analysis was generally not the central focus of these studies, it was mentioned in 26 out of 40 abstracts. Only a small subset of the published studies we surveyed provided a full list of significantly enriched functional categories. Several studies, however, reported in the main manuscript on a few significant GO terms appealing for interpretation (e.g. Ma *et al*., 2013). Readers are likely to be unaware that hundreds of other GO terms are equally enriched. Although not fundamental to the standard method, this problem is exacerbated by its unfailing production of large numbers of significantly enriched terms.

We were able to collect lists of differentially expressed (or otherwise flagged) miRNAs from 22 studies in humans and 7 studies in mice for analysis with our empirical algorithm (Supplementary Table S3). Where a study performed enrichment analysis for multiple miRNA lists, we arbitrarily selected one list per published study. In order to mirror the approaches in published studies as closely as possible, where each miRNA was analysed separately, we also applied the empirical algorithm in a single test of only one miRNA. We converted miRNA names to their current annotations, removing 10 miRNAs whose miRBase entries had been deleted since publication (Kozomara and Griffiths-Jones, 2011). For each input miRNA list, we ran our algorithm with miRanda predictions (Enright *et al*., 2003), biological process GO term annotations (Ashburner *et al*., 2000; Huang *et al*., 2009) and one million iterations of randomly selected miRNAs, generating empirical distributions of GO term target gene overlap for the specified numbers of input miRNAs and outputting *P*-values for the enrichment of GO terms.

After Benjamini–Hochberg correction for multiple testing (Benjamini and Hochberg, 1995), we observed an enrichment of any GO term in only 5 out of 22 human and 0 out of 7 mouse studies (Table 2). In contrast, all of the published studies reported multiple enriched functional categories. Although our aim is to provide a controlled comparison of standard and empirical methods, rather than to attempt to replicate the exact prediction and annotation methods of the previous studies, these results show that most functional categories reported to be enriched in the literature would not remain so after correction for bias.

**Table 2.** Results of empirical algorithm applied to published miRNA lists

References	Species	MicroRNA list extracted	Significant GO terms with basic empirical algorithm	Significant GO terms with multi-hit empirical algorithm
Arndt *et al.* (2009)	Human	1	0	0
Chartoumpekis *et al.* (2012)	Mouse	10	0	17
Chen *et al.* (2010)	Human	176	6017	1193
Chen *et al.* (2013b)	Human	15	0	6
Cheng *et al.* (2009)	Mouse	1	0	0
Collino *et al.* (2010)	Human	11	0	0
Davidson *et al.* (2010)	Human	1	0	0
Flavin *et al.* (2009)	Human	2	0	0
He *et al.* (2013)	Mouse	8	0	0
Hunter *et al.* (2008)	Human	1	0	0
Jiang *et al.* (2008)	Human	18	0	0
Keck-Wherley *et al.* (2011)	Mouse	12	0	0
Kraemer *et al.* (2013)	Human	9	0	0
Liang *et al.* (2013)	Mouse	37	0	0
Liu *et al.* (2010)	Mouse	27	0	1
Mizuguchi *et al.* (2011)	Human	3	0	0
Munch *et al.* (2013)	Human	10	0	22
Presneau and Eskandarpour (2013)	Human	5	0	0
Raponi *et al.* (2009)	Human	15	0	239
Romero-Cordoba *et al.* (2012)	Human	130	5573	206
Sanchez-Diaz *et al.* (2013)	Human	26	2502	28
Schonrock *et al.* (2010)	Mouse	1	0	0
Soh *et al.*, (2013)	Human	1	0	0
Sokolov *et al.* (2012)	Human	39	0	337
Tanic and Andrés (2013)	Human	46	3353	317
Wu *et al.* (2011)	Human	25	2480	138
Yan *et al.* (2012)	Human	1	0	0
Zhang *et al.* (2013)	Human	1	0	0
Ziats and Rennert (2013)	Human	25	0	0

We also investigated the use of the standard method in plant studies, using miRNA lists from four published rice articles as a test set (Supplementary Table S4). Following a typical analysis procedure, we ran our algorithm with these miRNA input lists, target prediction obtained using psRNATarget and GO annotations obtained from agriGO (Dai and Zhao, 2011; Du *et al*., 2010). We found significant enrichments in two out of the four input lists (Table 3), suggesting a similar general pattern to that in humans and mice.

**Table 3.** Results of empirical algorithm applied to published miRNA lists in rice studies

References	Species	MicroRNA list extracted	Significant GO terms with basic empirical algorithm
Abrouk *et al.* (2012)	Rice	69	34
Peng *et al.* (2011)	Rice	90	0
Wei *et al.* (2011)	Rice	68	0
Yi *et al.* (2013)	Rice	142	49

### 3.3 Robustness to prediction and annotation methods

Our results are robust to changes within the general framework of the standard method. Subsets of GO are often used in the literature. We therefore repeated all analyses using the filtered GO term annotation set *BP_FAT* downloaded from the DAVID Knowledgebase (Huang *et al*., 2009), with very similar results. As an alternative to biological process GO terms, we also used KEGG pathway annotations. Running the empirical algorithm with the published miRNA lists, 8 out of 29 miRNA lists produced at least one significantly enriched KEGG pathway. It is common to predict targets of miRNAs using several programs and to use the intersection set of their outputs. As an alternative to prediction by miRanda (Enright *et al*., 2003) alone, we used the intersection set of target predictions by PITA (Kertesz *et al*., 2007), DIANA-microT-v4 (Reczko *et al*., 2012) and seed matching using canonical seeds (Bartel, 2009). These downloaded prediction sets did not include all currently annotated miRNAs; in particular 134 of the miRNAs from input lists were missing and so had to be excluded. Using this prediction method, 3 of the 29 miRNA lists from published studies produced significantly enriched biological process GO terms. We also filtered target prediction sets to include only miRanda target loci supported by CLIP-seq data (see Section 2), similar to the miRFunction approach (Li *et al*., 2014). This had the effect of drastically reducing the number of predicted targets per miRNA. A similar pattern was observed, with no significant enrichments for 18 out of 22 human and 7 out of 7 mouse miRNA lists (Supplementary Table S2). The lack of enrichment is, therefore, robust to the number of predicted targets.

We tested whether our algorithm was able to detect functional enrichment when the input miRNAs were artificially selected for their targeting of a given process. We manually selected as an input set the eight miRNAs with the most predicted targets in the process ‘regulation of axonogenesis’ (GO:0050770). As expected, the algorithm found that the same GO term was significantly enriched, as well as other related and unrelated terms (Supplementary Table S5).

### 3.4 Testing for multi-hit convergence on processes

The standard method in its simplest form counts each gene once, whether it is targeted by one or many differentially expressed miRNAs, losing key information on functional convergence (Gusev, 2008; Lee *et al*., 2012). Filters on target gene sets or on output GO terms have been proposed previously to resolve this problem (Gusev, 2008; Hu *et al*., 2014). These filters require the proportion of miRNAs targeting a gene and the proportion of miRNAs with at least one target in a GO term to pass defined thresholds. Another alternative is to apply the statistical test on miRNA–mRNA pair connections (Lee *et al*., 2012). We therefore modified our algorithm to address this issue while maintaining our simple hypothesis testing approach and the principled comparison with the empirical distribution. The set of target genes for an input miRNA list was previously defined as the union of predicted target genes. In our modified algorithm, each gene is assigned a score for strength of interaction with miRNAs based on the total number of predicted binding sites, including multiple sites for the same miRNA. The score for a GO term is then the sum of gene scores for its members, divided by the total number of binding sites for the miRNAs. As above, we run one million iterations with randomly sampled miRNAs and compare the GO term scores for differentially expressed miRNAs with this empirical distribution.

Results from our modified algorithm applied to previously identified miRNA lists are shown in Table 2. Significant enrichments were found for all the input lists that had positive results for the original basic algorithm, albeit with more modest numbers of significant GO terms. Enrichments were also detected from six lists where previously they were not found.

## 4 Discussion

Our comparison of the hypergeometric and empirical distributions showed that certain functional categories are preferentially targeted by miRNAs, regardless of whether those miRNAs are differentially expressed in a biological state or not. It is not helpful to report a GO term as enriched for targets of differentially expressed miRNAs if an equally strong enrichment would be obtained for randomly picked miRNAs. This justifies an empirical sampling approach, which measures enrichment relative to other miRNAs, in comparison to the standard method, which does not take into account the background level of targeting of a GO term. There are several possible explanations for the phenomenon of preferential targeting by miRNAs, including bias in target prediction algorithms, similarities among seed sequences, correlations between genes that are regulated together and genuine preference for control of certain biological processes by different mechanisms. One clear source of bias is average 3′ UTR length of genes annotated to specific GO terms. When we use the *P*-value of the mean of the empirical distribution on the hypergeometric distribution (Table 1) as a measure for bias of a GO term, we observe a strong negative correlation with average 3′ UTR length of genes assigned to that GO term (Pearson's *r* = −0.36; *P* = 5.3 × 10^−^^287^). We also note that many GO terms were invariably returned together as enriched. This may reflect underlying correlations between targeting of processes as well as the hierarchical structure of the GO.

The simplest use of functional enrichment tests examines a set of genes with a common characteristic—for example, a set of differentially expressed genes, or a set of genes with particular genomic properties. The test described here is subtly different: a set of miRNAs is defined by differential expression and that set is one step removed (by miRNA target prediction) from the set of genes whose functional enrichment is tested. The bias in the underlying expected distribution of functional categories comes from the process of linking miRNAs with their target genes. While we have examined only a specific use of the functional enrichment test, similar biases may affect other genomic enrichment tests (Slowikowski *et al*., 2014). For example, ChIP-seq identification of transcription factor-binding sites followed by functional enrichment of the target gene set is analogous to the analyses described here. Further investigation is therefore required to determine the appropriateness of the hypergeometric distribution for other types of functional enrichment studies.

In our literature survey, we identified 44 journal articles that used the standard method. However, our list was not exhaustive and excluded studies with relatively minor deviations from the standard method, such as those that implemented more complex filters for selection of target genes (Cho *et al*., 2013) and those that performed tests based on combined targeting by miRNAs (Lee *et al*., 2012). Altogether, we estimate that hundreds of published articles are likely to be affected by the bias described here. In the application of the empirical algorithm to miRNA lists from these studies, we do not attempt to directly replicate methods used and instead show a pattern in results that strongly suggests that most reported enrichments would not be found with correction for bias. We also do not directly compare the significant enrichments output by our algorithm with those reported, as the specific types of functional categories assayed, such as GO term collections assembled by DAVID (Huang *et al*., 2009), vary greatly and full lists of significant terms are rarely published.

Our results do not imply that differentially expressed miRNAs do not converge on functions of interest. It is notable that the number of significantly enriched GO terms increased with the number of miRNAs input to our algorithm. This may be because noise dominates for smaller lists, whereas a larger number of input miRNAs provides more signal of convergence on a process. While modest enrichment of a function for a single miRNA is undetectable, the combination of many small enrichments for a larger collection of miRNAs passes significance thresholds. Five of the largest input miRNA lists (Chen *et al*., 2010; Romero-Cordoba *et al*., 2012; Sanchez-Diaz *et al*., 2013; Tanic and Andrés, 2013; Wu *et al*., 2011) had extremely convergent miRNA target sets (for example (Chen *et al*., 2010) had a predicted target set significantly smaller than expected [*P* < 10^−^^5^]). Convergent target sets mean that any GO term hit by chance will contain a higher proportion of the target set, causing more significant GO terms to be returned. There have been other proposals to try to harness the convergence of miRNAs and to improve enrichment analysis. The miRSystem tool gives a *P*-value based on the tendency for the standard method to consistently find the same significant GO terms (Lu *et al*., 2012). By comparing the order of enrichment for a new sample with the order for random miRNAs, outstanding changes can be identified. Although use of a pre-computed distribution limits the flexibility of miRSystem, the approach escapes the problem of bias reported here (Lu *et al*., 2012). In general, however, there remains an unfulfilled need for more powerful and accurate bioinformatic tools to link miRNAs to functions.

## 5 Conclusion

We have highlighted critical problems with the most common general approach to functional enrichment analysis of miRNA target genes. We have shown that testing with the hypergeometric distribution sampling from all GO annotated genes in the genome is inappropriate. Our literature survey showed that a large number of studies reported significant results that are unlikely to stand after correction for the bias in the distribution of targets of randomly sampled miRNAs. We believe that our results provide a strong argument against continued use of the standard method.

## Supplementary Material

Supplementary Data
