# Supplementary material for: Bias in microRNA functional enrichment analysis
Source: Bioinformatics. 2015 Jan 20;31(10):1592–8. doi: 10.1093/bioinformatics/btv023 (PMC4426843; doi:10.1093/bioinformatics/btv023)
Supplement: Supplementary Data [file supp_31_10_1592__index.html]

Bias in microRNA functional enrichment analysis — Bias in microRNA functional enrichment analysis — Bias in microRNA functional enrichment analysis — Supplementary Data 

# Bias in microRNA functional enrichment analysis

## Supplementary Data

files

**Files in this Data Supplement:**

- Supplementary Data - xls file
- Supplementary Data - xls file
- Supplementary Data - xls file
- Supplementary Data - xls file
- Supplementary Data - xls file
